# PKCζ Mediates Breakdown of Outer Blood-Retinal Barriers in Diabetic Retinopathy

**DOI:** 10.1371/journal.pone.0081600

**Published:** 2013-11-29

**Authors:** Samy Omri, Francine Behar-Cohen, Pierre-Raphaël Rothschild, Emmanuelle Gélizé, Laurent Jonet, Jean Claude Jeanny, Boubaker Omri, Patricia Crisanti

**Affiliations:** 1 Institut National de la Santé et de la Recherche Médicale, U872, Paris, France; 2 Université Paris Descartes, UMRS 872, Paris, France; 3 Centre de Recherche des Cordeliers, UPMC-Paris6, UMRS 872, Paris, France; 4 Hôtel-Dieu Hospital, AP-HP, Paris, France; Eye Hospital, Charité, Germany

## Abstract

**Aims/hypothesis:**

Diabetic macular edema represents the main cause of visual loss in diabetic retinopathy. Besides inner blood retinal barrier breakdown, the role of the outer blood retinal barrier breakdown has been poorly analyzed. We characterized the structural and molecular alterations of the outer blood retinal barrier during the time course of diabetes, focusing on PKCζ, a critical protein for tight junction assembly, known to be overactivated by hyperglycemia.

**Methods:**

Studies were conducted on a type2 diabetes Goto-Kakizaki rat model. PKCζ level and subcellular localization were assessed by immunoblotting and immunohistochemistry. Cell death was detected by TUNEL assays. PKCζ level on specific layers was assessed by laser microdissection followed by Western blotting. The functional role of PKCζ was then evaluated *in vivo*, using intraocular administration of its specific inhibitor.

**Results:**

PKCζ was localized in tight junction protein complexes of the retinal pigment epithelium and in photoreceptors inner segments. Strikingly, in outer segment PKCζ staining was restricted to cone photoreceptors. Short-term hyperglycemia induced activation and delocalization of PKCζ from both retinal pigment epithelium junctions and cone outer segment. Outer blood retinal barrier disruption and photoreceptor cone degeneration characterized long-term hyperglycemia. *In vivo*, reduction of PKCζ overactivation using a specific inhibitor, restored its tight-junction localization and not only improved the outer blood retinal barrier, but also reduced photoreceptor cell-death.

**Conclusions:**

In the retina, hyperglycemia induced overactivation of PKCζ is associated with outer blood retinal barrier breakdown and photoreceptor degeneration. *In vivo*, short-term inhibition of PKCζ restores the outer barrier structure and reduces photoreceptor cell death, identifying PKCζ as a potential target for early and underestimated diabetes-induced retinal pathology.

## Introduction

Diabetic retinopathy (DR) is one of the most severe complication of diabetes and the leading cause of visual loss among western working-age adults [1], [2]. Visual impairment results mostly from macular edema (ME), defined as fluid accumulation in/or under the macula, which is the specialized retinal area responsible for visual acuity [3], [4]. Retinal edema results from an imbalance between fluid entry and fluid withdrawal, but the precise mechanisms of ME are still elusive. Fluid entry is controlled by the inner blood retinal barrier (BRB) made of TJ between the endothelial cells of the retinal vasculature together with a complex network of glial components. The retinal pigment epithelium (RPE) is a monolayer of pigmented cells with tight-junctions that control mostly fluid exit from the sub-retinal space through the choroid. The apical side of RPE faces the photoreceptor outer segments of the neuroretina, and the basolateral side lies on Bruch's membrane, which separates the RPE from the underlying fenestrated endothelium of the choriocapillaris [5]. We have recently evidenced that tight-like junctions containing occludin are present also at the OLM, mainly between Müller glial cells and cones, the only photoreceptors of the macula [6]. Among the molecules involved in TJ formation and maintenance, the critical role of the PAR6/PKCζ/PAR3 scaffold complex in cell polarity has been extensively studied [7]–[13]. Any protein loss of this complex leads to mislocalization of the others [14]. PKCζ also phosphorylates occludin and thereby promotes assembly of TJ [15] and it was shown that hyperglycemia modulates its activity.[16], [17]

Whilst extensive work has focused on hyperglycemia-induced inner BRB breakdown, much less attention has been carried towards the effect of hyperglycemia on the outer retina.

The aim of our work was to evaluate in a model of type 2 non-obese diabetic rat, the effects of hyperglycemia on the outer retinal barriers components and their consequences on the retinal structure. We observed that rupture of the outer retinal barriers is an early hyperglycemia-induced event and that it is associated to photoreceptor cell death. Moreover, we identified PKCζ as a potential regulatory target in these early events.

## Materials and Methods

### Animals

The animals used in this work were treated in accordance with the Association for Research in Vision and Ophthalmology (ARVO) statement. Experimental procedures were submitted and approved by the local ethics committee of Paris Descartes University.

Goto-Kakizaki (GK) rats (Taconic Europe, Denmark), a Wistar non-obese model of Non-Insulin Dependent type 2 Diabetes were studied at 6 and 12 months of age. Non-fasting blood glucose level was measured using Accutrend GC and Accu-check compact equipments (Roche). A plasma glucose level>250 mg/dl (14 mmol/l) defined the diabetic status. In contrast to control Wistar rats, GK rats develop hyperglycemia at around 14 weeks of age and remain diabetic thereafter (Table 1). Controls were selected from age-matched rats with plasma glucose levels < 150 mg/dl (8.5 mmol/l).

**Table 1:** Characteristics of the study animals.

	Control		Diabetes	
Age (months)	6	12	6	12
Number of animals	24	24	24	24
Weight (g ± SEM)	212±10	341±7	305±15	387±15
Blood Glucose level (mmol/l ± SEM)	6.33±0.67	7.44±0.28	19.33±0.56^a^	22.39±0.67^a^

a: diabetes versus controls, P value<0.05.

### Immunohistochemistry on flatmounted neuroretina or RPE/choroid

After sacrifice, rat eyes were enucleated, fixed in 4% paraformaldehyde (PFA) for 15 min at room temperature and sectioned at the limbus; the anterior segments were discarded. Neuroretinas and RPE/choroids were dissected and fixed separately for an additional 15 min in acetone at −20°C. Specimens were then incubated overnight at 4°C with primary antibodies diluted in PBS supplemented with 10% fetal calf serum (FCS) and 0.1% Triton X-100. Primary antibodies used were: rabbit polyclonal anti-occludin (71–1500) (Zymed, San Francisco, CA, USA) (dilution 1∶200); mouse monoclonal anti-PKCζ (ab57432) raised against amino acid 165 to 255, specific of the N-terminal region of the PKCζ; rabbit polyclonal anti-Partitioning-defective protein 3 (PAR3) (ab64840) (Abcam, Cambridge, UK) (dilution 1∶400); anti-Partitioning-defective protein 6 (PAR6) (H-90) (sc-25525) (dilution 1∶400) and anti-β-tubulin (D-10) (sc-5274) (Santa Cruz Biotechnology, Inc, Santa Cruz, CA, USA) (dilution 1∶400); Goat polyclonal Blue-sensitive opsin (SC 14363) (Santa Cruz Biotechnology, Inc, Santa Cruz, CA, USA) (dilution 1∶200); rabbit polyclonal anti- P-PKCζ (sc-12894-R) raised against the phospho-threonin 410 amino acid (p-T410) of the PKCζ (dilution 1∶400), anti-P65-NF-κB (sc-33039-R) (serine 311-R), (Santa Cruz Biotechnology, Inc, Santa Cruz, CA, USA) (dilution 1∶200).

The corresponding Alexa secondary antibodies (Invitrogen life technology Carlsbad) were used to reveal the primary antibodies (dilution 1∶250), and sections were counterstained with 4′, 6-diamino-2-phenylindol (DAPI) (Sigma Aldrich St. Louis, MO USA) (dilution 1∶5000).

Sections and flatmounts were viewed with a fluorescence microscope (BX51; Olympus, Rungis, France) and confocal microscope (LSM 510 laser scanning microscope Zeiss, Carl Zeiss, Le Pecq, France). Images were then processed by Photoshop software for preparation of the final images.

The immunostaining procedure was repeated at least 3 times to seek for consistency of the positive results. Negative controls were obtained by staining procedures that omitted the primary antibody.

### Laser microdissection of specific retina regions

The OLM region was extracted from the neuroretina by laser microdissection of the outer retina on frozen sections using a Leica AS LMD system (Leica, Solms, Germany) as previously described by Burbach and colleagues [18]. Protein extracts from at least 30 sections of 40 μm per eye were used for immunoblot analysis.

### Tunel assay

Cell apoptosis was assessed with a terminal dUTP nick-end labeling (TUNEL) kit (Roche, Indianapolis, IN, USA)) on retina sections. Staining conditions were as specified in the manufacturer's instructions.

### Western blotting

Protein extracts from RPE/choroid and from laser microdissected OLM regions were obtained from diabetic rats of 6 and 12 month of age and from age-matched controls. Proteins were homogenized in a lysis buffer (10 mM Tris-HCl, pH 7.5, 1 mM EDTA, 1 mM EGTA, 150 mM NaCl, 0.5% Nonidet P40, 1% Triton X-100, β-mercaptoethanol) containing a protease inhibitor cocktail (Roche, France). Protein concentration was determined using a Bradford assay. Proteins (40–50 µg) were subjected to SDS-PAGE in a 12% sodium dodecyl sulfate-polyacrylamide gel electrophoresis, and electroblotted onto nitrocellulose membranes (Schleicher and Schuell BioScience, Dassel, Germany). Membranes were then incubated with: mouse monoclonal anti-PKCζ (ab57432) raised against amino acid 165 to 255, specific of the N-terminal region of the PKCζ (Abcam, Cambridge, UK) (dilution 1∶400); rabbit polyclonal anti PKCζ–P (sc-12894-R) raised against the phospho-threonine 410 amino acid (p-T410) of the PKCζ (dilution 1∶400), anti-Partitioning-defective protein 6 (H-90) (sc-25525) (dilution 1∶200) and anti-β-tubulin (D-10) (sc-5274) (Santa Cruz Biotechnology, Inc, Santa Cruz, CA, USA) (dilution 1∶400). Rabbit polyclonal anti-Partitioning-defective protein 3 (07-330) (EMD Millipore Life Science division of Merck KGaA of Darmstadt, Germany)(dilution 2 µ/ml). Then, membranes were incubated with the corresponding peroxidase-conjugated F(ab)2 fragment (Caltag, Burlingame, Canada) (dilution1∶4000) secondary antibodies. Immunoreactive bands were detected with the ECL Western blotting Detection Reagents Kit (Thermo scientific inc, IL, USA). The relative abundance of individual proteins identified was quantified by scanning densitometry. For the phosphorylated PKCζ (PKCζ -P) the relative band intensity was calculated in comparison to the non phosphorylated PKCζ after densitometry analysis (Adobe photoshop software).

### Intravitreal injection of a specific PKCζ inhibitor

Rats of 6 months of age were anesthetized with an intraperitoneal injection of pentobarbital (40 mg/kg Nembutal, Abbot, Saint-Remy sur Avre, France). Pupils were dilated with the instillation of a 1 drop of 5% tropicamide (Ciba Vision, Toulouse, France), and locally anesthetized with a 1 drop of 1% tetracaïne (Ciba Vision, Toulouse, France). Injections, into the vitreous of a 3 μl sterile pyrogen-free saline containing either a PKCζ specific inhibitory peptide (n = 5) (myr-DIYRRGARRWRKL, ref. 539624 Lot B42131, Calbiochem) or a sham (n = 5), were performed as previously described [19]–[21] through sterile syringes with a 30–gauge needle (Microfine: Becton Dickinson, Meylan, France). The injection procedure was monitored under a surgical microscope.

### Semithin sections

Eyes were fixed for 1 hour in 2.5% glutaraldehyde in cacodylate buffer (0.1 mol/L, pH 7.4). Eyes were dissected, fixed for 3 hours, postfixed in 1% osmium tetroxide in cacodylate buffer, and dehydrated in graduated ethanol solutions. Samples were included in epoxy resin and oriented. Semithin sections (1 μm), ultra microtome Reichert Ultracut E [Leica], were stained by toluidine blue.

### Fluorescence quantification

Fluorescent images were scanned at the same magnification and exposure conditions. Quantification was performed using the Image J software (National Institutes of Health, Bethseda, MD, USA). First, the total area of the section was measured, and PKCζ specific areas were determined. The percentage of PKCζ was then determined.

### Statistics

For continuous variables, the mean ± SEM were provided. Comparisons were performed by the non parametric, Mann–Whitney test (Prism software version 4.0c; GraphPad Software, San Diego, CA). Statistical significance was accepted for P value <0.05.

## Results

### Hyperglycemia-induced changes in PKCζ activity and distribution in RPE cells is associated with barrier integrity alteration

PKCζ level was evaluated by Western blotting on RPE/choroid protein extracts and its cellular localization was analyzed by immunohistochemistry on RPE/choroid flat mounts. Two PKCζ antibodies were used: - one directed against the N-terminal (N-ter) domain, - the other against the activated form of PKCζ, phosphorylated at the Thr 410 residue (p-T410).

**Figure 1:**
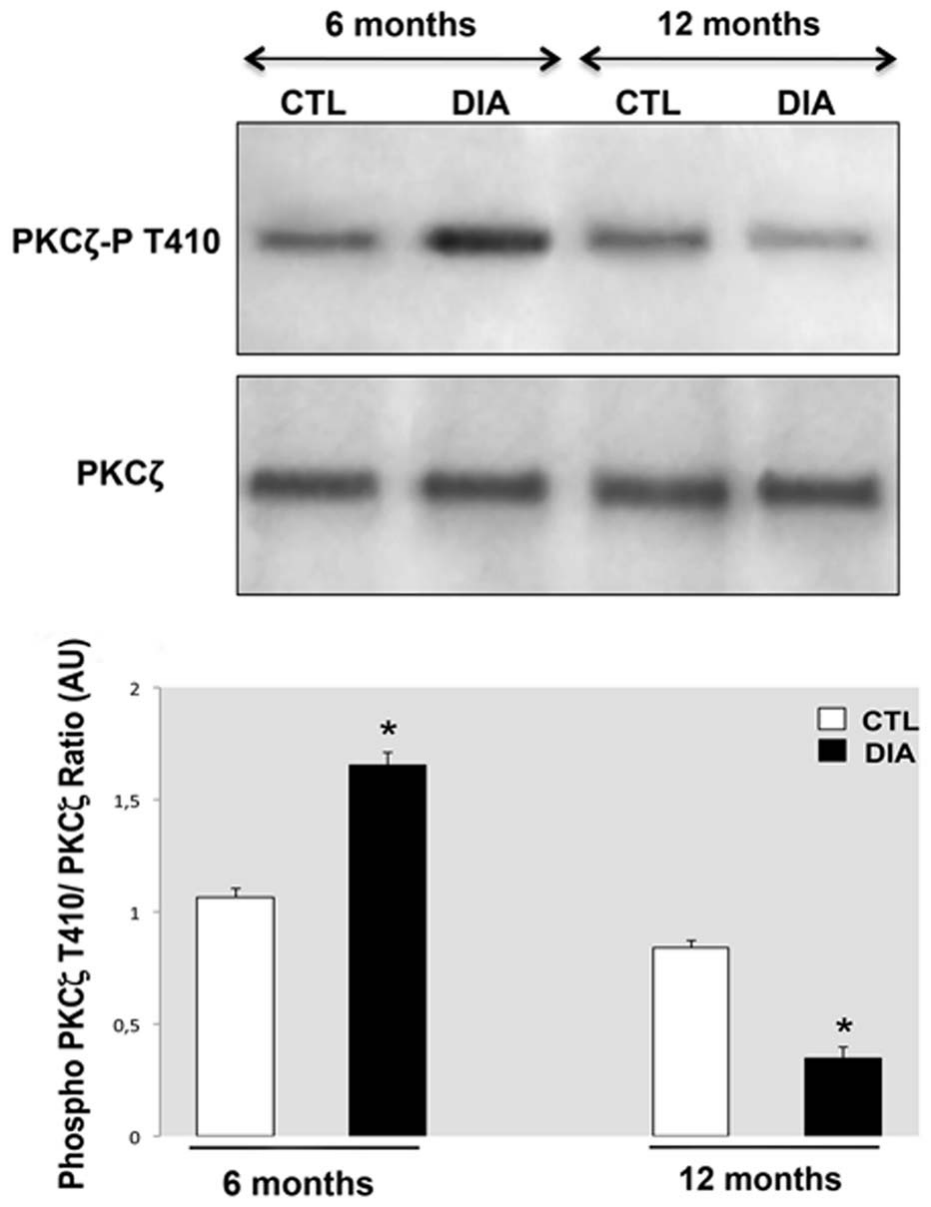
Diabetes stage-specific retinal pigment epithelium (RPE) level of PKCζ and its activated phosphorylated form PKCζ-P T410. No significant changes were observed for PKCζ level at any stage between control and diabetic conditions. To the contrary, PKCζ-P T410 immunoreactivity was significantly (*P<0.05, Mann–Whitney test) increased (by around 40%) at 6 months and then decreased (by around 60%) at 12 months of diabetes compared to controls.

Total PKC**ζ** levels did not differ significantly in diabetic RPE as compared to the control non-diabetic rats of 6 and 12 months of age (Fig 1), but in diabetic rats, its phosphorylated (p-T410) active form increased (by around 40%) at 6 months of age and then significantly decreased (by around 60%) at 12 months of age (Fig 1). There is therefore a biphasic activation state of PKCζ in the time course of diabetic retinopathy.

**Figure 2:**
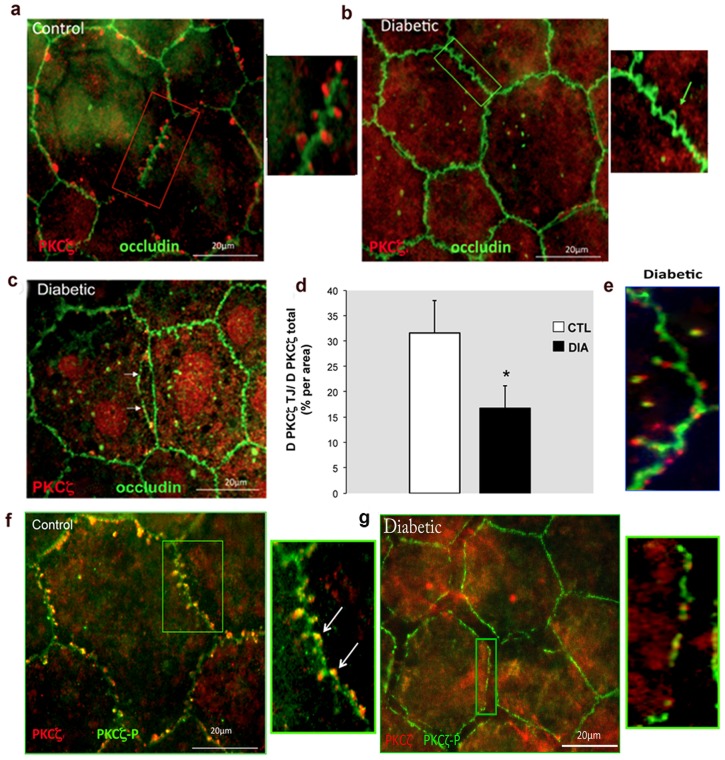
Immunolocalization of PKCζ and occludin in retinal pigment epithelium (RPE) flatmounts. In 6-month-old controls, PKCζ staining (red) appeared as focal spots within the loops depicted by occludin (green) protrusions (a). In age-matched diabetic conditions, no PKCζ labeling (box and green arrow) was observed (b). After 12 month of diabetes marked disruption of intercellular junctions (arrows) was evidenced (c). At this stage the PKCζ at the junctions (TJ)/total amount of PKCζ ratio was significantly decreased for diabetic conditions (Image J software, National Institutes of Health, Bethesda, MD) (d) and occludin internalization from the cell membrane into the cytoplasm was found (e). The colocalization of PKCζ (red) and PKCζ-P (green) found in 12-month-old controls (f) was not present in age-matched diabetics (g). Furthermore the activated PKCζ-P form exhibited a discontinuous staining along the cell membrane (g).

Immunolocalization of total PKCζ in RPE cells of control rats was found to be highly organized and appeared as focal spots within the loops depicted by occludin protrusions from both sides of the continuous occludin labeling found at the cell–cell contacts (Fig 2a). Conversely, in RPE cells from 6-month-old diabetic rats, the PKCζ staining was either barely or not detectable associated to the TJ but instead a diffuse staining of PKCζ was observed in the cytoplasma (Fig 2b). At this stage, the outer BRB did not seem to be significantly disrupted. At 12 months of age, in diabetic RPE, PKCζ and occludin could be observed in the nuclei of cells, while marked disruptions of the tight junctions with buttonhole formation could be observed (Fig 2c). At this time point (12 months), to better localize the phosphorylated active PKCζ form as compared to the total PKCζ, we used two distinct antibodies. This allowed identify the phosphorylated form (PKCζ-P) evenly associated to the junction at the membrane in control RPE (Fig 2f). In RPE from diabetic rats, the phosphorylated form seemed reduced and weekly associated to the membrane, its distribution pattern was also markedly discontinued and RPE intercellular junctions exhibited various degrees of disruption (Figure 2g). To better illustrate this change, we quantified the PKCζ at the TJ/total amount of PKCζ ratio, showing indeed a significant reduction in diabetic rats (Fig 2d). However, a diffuse labeling of PKCζ (Fig 2g) well correlated with the stable levels of total PKCζ during the time course of diabetes (as shown above in Figure 1).

Furthermore, we found that occludin was also delocalized from the membrane further contributing to RPE TJ disruption (Fig 2e and 2c). Of importance, we confirmed that PKCζ localization pattern in non-diabetic, non human primates flatmount RPE cells was similar to that found in control rats (data not shown).

### Hyperglycemia-induced BRB TJ disruption through destabilization of the PAR3/PAR6 PKCζ-associated complex

**Figure 3:**
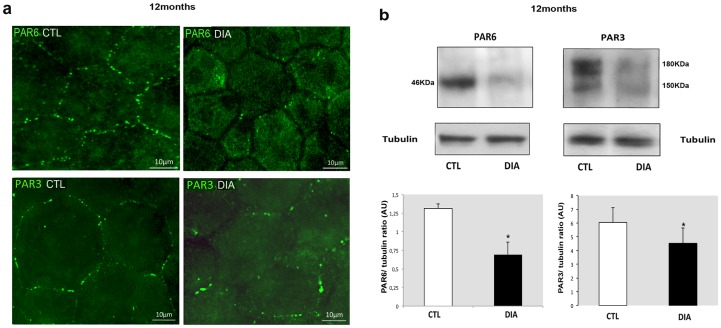
Diabetes destabilizes and down regulates PAR3/PAR6 PKCζ-associated protein complex. a) Upper panels: In 12-month-old diabetic rats (DIA) PAR6 distribution in flatmount RPE showed a clear cytoplasmic relocation of PAR6 compared to age-matched control rats (CTL) where PAR6 appeared as relative regular punctiform staining at the TJ levels. The same applied to PAR3 staining distribution (Lower panels). b) PAR6 and PAR3 immunoblotting on RPE cell extracts from 12-month-old rats showed a significant decrease of both protein levels in diabetic conditions (statistical analysis was performed on the PAR3 180 KDa isoform).

Because both PAR3 and PAR6 proteins are known to interact with PKCζ at the tight junction protein complexes, PAR3 and PAR 6 localization was investigated by immunohistochemistry and quantified by Western blotting. Compared to controls, the 12-month-old diabetic rat RPE cells, showed decreased immunostaining of both PAR3 and PAR6 at the tight junction structures (Fig 3a). Furthermore, in RPE cells from 12-month-old diabetic rats, both PAR3 and PAR6 protein levels were also significantly decreased (Fig 3b).

Altogether, these results suggest that after 12 months of diabetes duration, PKCζ down regulation contributes to an imbalance of the TJ protein complex composition and/or distribution that may initiate RPE junction destabilization.

### Beneficial effect of intravitreal injection of a PKCζ inhibitor on diabetic RPE barrier

**Figure 4:**
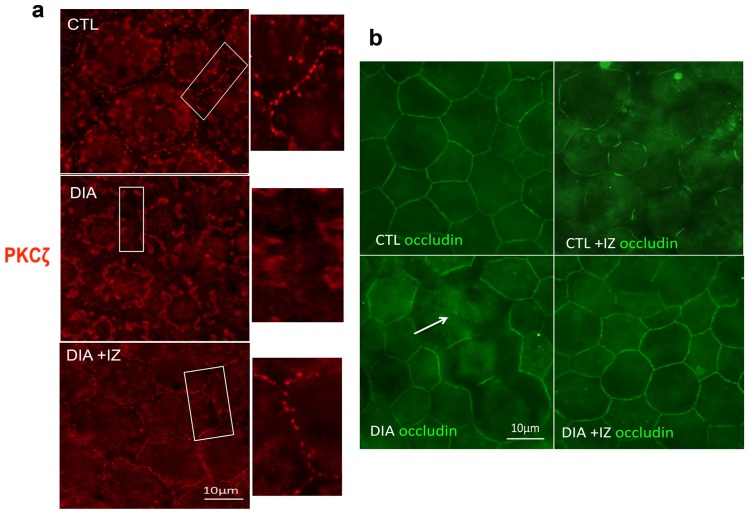
PKCζ inhibition in 6-month-old diabetic rats restores PKCζ localization and RPE junction structure. After a single intravitreal injection, PKCζ staining (red) was restored and relocated at the tight junctions (DIA+IZ) in RPE flatmounts (a). Diabetic treated rats (DIA+IZ) exhibited relocalization of occludin staining at the membrane level and restoration of the intercellular junctions (b). Of importance PKCζ inhibition in controls induced marked alteration of occludin staining at the membrane, thereby confirming the critical role of this kinase in TJ formation and maintenance in normal conditions (b).

The above described results suggest that PKCζ overactivation plays a role in the disruption of TJ in RPE during diabetes. To ascertain this hypothesis, both control and diabetic rats at 6 months of age were given a single intravitreal injection of a PKC inhibitor (IZ). Rats were then sacrificed and processed within 48 hours. In diabetic treated eyes (IZ), immunostaining on flatmounted RPE showed restoration of PKCζ organization within the occludin loops, similar to what is observed in control (CTL) animals (Fig 4a). Diabetic sham treated eyes (DIA) exhibited either faint or no PKCζ staining at the junction (Fig 4a). Similarly, occludin staining was altered in 6-month-old sham treated diabetic rats (DIA) and was restored by the treatment (DIA+IZ) (Fig 4b). To note, controls treated by the PKCζ inhibitor (CTL+IZ) showed a marked decrease in the recruitment of occludin at the membrane thereby confirming the role of this kinase in TJ maintenance in normal conditions (Fig 4b).

### Diabetes induces retinal morphological changes and cone photoreceptor degeneration

**Figure 5:**
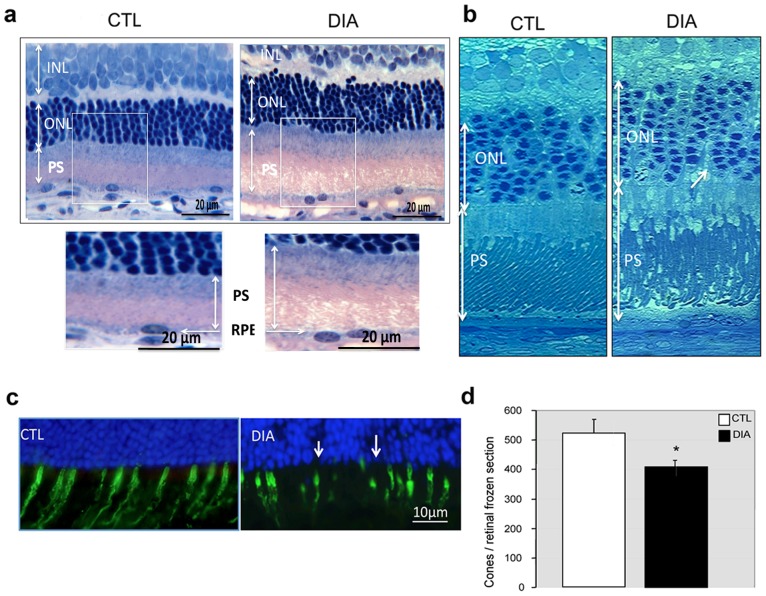
Diabetes induces outer retinal edema and loss of cones photoreceptors. Diabetic retina (DIA) demonstrated increased extracellular spaces within the outer nuclear layer (ONL) and photoreceptor segment disorganization, consistent with an edematous aspect of the outer retina (Historesin (a) and semithin sections (b)). Peanut agglutinin labeling, a specific marker of cone extracellular matrix, evidenced a marked decreased in cell cone density (c). Whole retina quantification of cones evaluated the net loss to be around 20% (d) as compared to controls (p = 0.002, Mann–Whitney test).

Because PKCζ is also tightly associated with junctions at the outer limiting membrane and contributes to its integrity, we first evaluated potential structural changes associated with diabetes in the outer retina. In 12-month-old diabetic rats, the outer retina appeared swollen (Fig 5a) with increased spaces between nuclei of photoreceptors (Fig 5b, white arrow). The outer segments of photoreceptors seemed also disorganized (Fig 5b) and cone cell density measurement by peanut agglutinin labeling, a specific marker of cone extracellular matrix, evidenced a 20% net loss of cone photoreceptors in diabetic retinas as compared to controls. (Fig 5c and 5d).

### Diabetes reduces PKCζ distribution in cone outer segments and at the outer limiting membrane together with structure integrity alterations in the outer neuroretina

**Figure 6:**
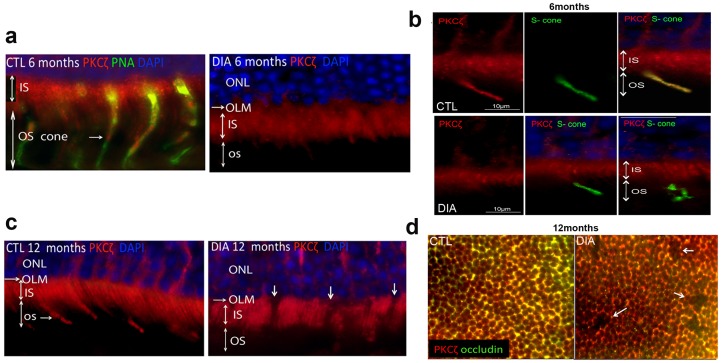
Alteration of PKCζ distribution in the outer retina is associated with cone outer segment and OLM disruption in diabetic rats. PKCζ staining was located in inner segments (IS) of rod and cones and exclusively in cone OS as evidenced by PKCζ-PNA double labeling in 6-month-old control rats (a). In 6-month-old diabetic rats, no OS staining was detected (a). Furthermore PKCζ staining (red) was lost in S-cones, specifically marked by Blue opsin staining (green), as compared to controls. Some S-cone OS also showed marked structural alterations, suggesting early photoreceptor degeneration (b). In 12-month-old diabetic rats OLM discontinuity (arrows) was evidenced (c). OLM tight-junction disruption was further confirmed on retinal flatmounts by an occludin/PKCζ double staining (d, white arrows).

The outer limiting membrane (OLM) is constituted by tight junctions between retinal Müller glial cells and photoreceptors. Occludin along with PKCζ, are hallmark proteins of TJ. In control retina, we observed indeed that PKCζ was expressed at the OLM but it was also expressed in the inner segments of photoreceptors and in inner and outer segments of cone photoreceptors, as indicated with PNA staining of cones co labeled with PKCζ (Fig 6a). Thus outer segments of photoreceptors stained with PKCζ were only of the cone type as shown by the PNA staining (Fig 6a). In diabetic rats, as early as 6 months of age, the cone outer segment PKCζ staining was lost (Fig 6a). We focused our investigations on the S-cone photoreceptor subset (or blue sensitive/short-wavelength cones) that have been reported to be the most susceptible to degeneration during diabetic retinopathy in humans [22]–[24]. The PKCζ/blue opsin OS co-staining found on retinal sections of 6-month-old control rats was lost in diabetic conditions (Fig 6b). Furthermore a significant disruption of some S-cone OS was observed suggesting early photoreceptor degeneration (Fig 6b).

At 12 months of diabetes, in addition to the changes observed at 6 months, discontinuities are observed at the OLM (Figure 6 c, arrows). To further assess TJ disruptions at the OLM level we performed a PKCζ/occludin co-staining on flatmounted retina from 12-months-old rats. We confirmed that the highly organized honeycomb pattern of the OLM TJ found around photoreceptor inner segments were markedly disrupted in diabetic rats (Fig 6d, white arrows). Indeed, we evidenced a loss of OLM integrity with reduced PKCζ/occludin co-staining in diabetic rats.

### PKCζ specific inhibitor partially restores photoreceptor alteration

To ascertain whether PKCζ overactivation was associated with its delocalization from cone outer segments in diabetic retinas we performed intravitreal injections of a PKC**ζ** inhibitor in 6-month-old diabetic rats and in age-matched controls. After 48 hours, rats were sacrificed and processed.

**Figure 7:**
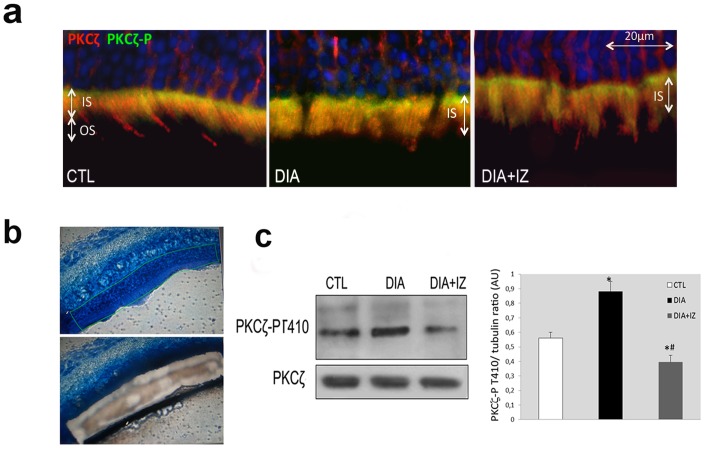
*In-vivo* PKCζ inhibition in 6-month-old-diabetic rats partially restores PKCζ staining pattern in cone outer segments. The phosphorylated active form PKCζ-P (green) was found in IS of both control (CTL) and diabetic (DIA) rats (a). Diabetic inhibitor (DIA+IZ) treated rats showed partial restoration of OS PKCζ (red) staining (a). We performed laser micro dissection of the photoreceptor layer on cryosections (b) to study layer-specific protein level by Western blotting analysis. The PKCζ-P immunoreactivity increase found in diabetic rats (DIA) was partially restored in treated rats (DIA+IZ).

Using two antibodies directed against PKCζ and its activated counterpart, the PKCζ-P, we could observe as for RPE cells, that at 6 months of diabetes (DIA), the active PKCζ-P form is increased and located in the inner segment of photoreceptor cells. Furthermore, PKCζ was delocalized from the cone OS to cone IS (Fig 7a). Whilst the total levels of PKCζ may not have changed, its distribution and phosphorylation was significantly modified in diabetic conditions.

Layer-specific protein level analysis, using a laser microdissection technique (Fig 7b) showed a stable protein level for PKCζ but a marked increase (by around 30%) of its activated form (Fig 7c) in diabetic conditions (DIA). These findings are consistent with a delocalization of PKCζ from the cone outer segments to the inner segments and an increase of its phosphorylated form in IS.

Intravitreal injection of a PKCζ inhibitor (DIA+IZ) restored, though partially, PKCζ immunolocalization in cone outer segments (Fig 7a) and PKCζ activation level (Fig 7c).

### Diabetes induces cone photoreceptor degeneration through NF-κB pathway

Hyperglycemia and subsequent oxidative stress and/or inflammation have been well documented in DR [25]. Besides PKCζ activation, these processes also involve NF-κB activation. Moreover PKCζ is required for NF-κB-mediated cell death by a specific phosphorylation at Ser 311 of its P65 subunit (P65-P) that finally results in both a nuclear translocation and activation of NF-κB. We therefore studied the implication of this pathway in photoreceptor degeneration in our model. These studies were performed on retinal cryosections from 6-month-old rats taking advantage of a specific antibody directed against this serine 311 residue. TUNEL assay was performed to asses photoreceptor cell-death.

**Figure 8:**
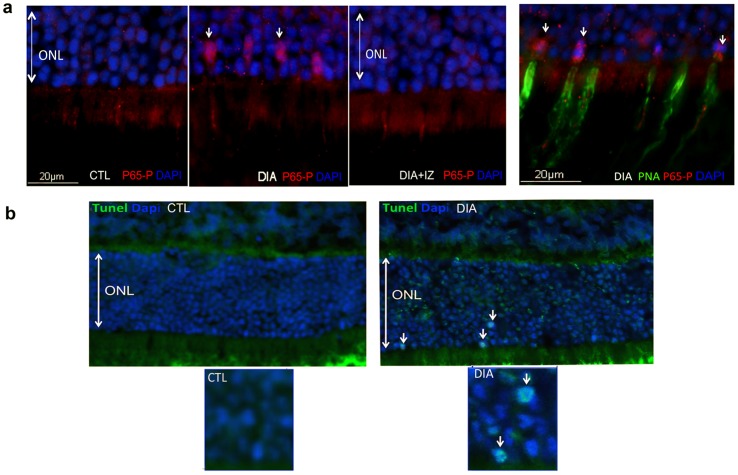
PKCζ specifically regulates NF-κB signaling pathway which participates to diabetes-induced cone photoreceptor degeneration. In 6-month-old rat cryosections, the p65-P subunit of NF-κB (red) was only detected in diabetic (DIA) conditions in some nuclei of the outer nuclear layer (*arrows*) and not in controls (CTL) or treated rats (DIA+IZ). The triple staining with addition of PNA (green), a specific cone marker, confirmed that the nuclear translocation p65-P subunit of NF-κB exclusively occurred in cones (a). In age-matched rat cryosections, TUNEL assay confirmed apoptosis of photoreceptors in diabetic (DIA) conditions (b).

A marked nuclear NF-κB P65-P subunit staining was detected in diabetic retinas (DIA, white arrows) as compared to controls (CTL) (Fig 8a). Strikingly this nuclear staining was selectively observed in cones as shown by the double labeling P65-P and PNA, a selective marker of cone extra cellular matrix. Furthermore, after PKCζ inhibitor treatment (DIA+IZ), no P65-P staining was observed in the outer nuclear layer (Fig8a).

TUNEL assay experiments performed at this disease-stage, showed rare (about 4% of photoreceptors) but reproducible TUNEL staining within some nuclei of the outer nuclear layer (ONL) of diabetic retinas (Fig 8b, white arrows) consistent with photoreceptor cell death through apoptosis. No significant positive TUNEL staining was observed either in control retinas or in diabetic treated retinas (data not shown). The scarce positive TUNEL staining found in cells undergoing death through apoptosis, could be explained by well-known short-lived nuclei TUNEL-positive status.

## Discussion

Retinal microvascular alterations and inner blood retinal barrier breakdown have been the focus of most of the research conducted so far in the field of diabetic retinopathy. Although a growing body of evidence suggest a role for the outer retinal barrier in DR, the literature deciphering the mechanism from a basic standpoint are still limited [5], [26], [27]. Herein we address the implication of the overlooked outer barriers in diabetic retinopathy in a rat model of type2 diabetes. We have recently shown that the OLM is not only formed by adherent junctions between glial Müller cells and photoreceptors but also contains heterotypic tight-junctions, suggesting that the OLM should be considered as part of the outer retinal barrier [6]. However, what regulates this barrier and what are the consequences of OLM barrier function alteration in the course of diabetes has not been explored.

Among the tight-junction protein complex, essential for barrier formation and maintenance, the critical role of PAR6/PKCζ/PAR3 scaffold has been extensively studied in different systems. The loss of any of these proteins leads to mislocalization of the others [18]. But, beyond its interaction with PAR3 and PAR6, PKCζ also phosphorylates occludin and thereby promotes tight-junction assembly, identifying PKCζ as a key player in the regulation of tight-junction function integrity [15].

In the retina of diabetic rats, much before any microvascular changes, we found that hyperglycemia induced PKCζ overactivation and dramatic localization changes of the protein that preceded outer BRB alteration and photoreceptor degeneration. PKCζ immunostaining in inner segments of both cone and rod photoreceptors was consistent with previous reports.[28], [29] However, we also observed that PKCζ labeling restricted to cone photoreceptors outer segments, was lost by short-term diabetes and associated to alteration of cone structure. Long-term diabetes was associated with photoreceptor degeneration involving NF-KB activation. We have therefore identified PKCζ as a potential specific and early regulator of cone survival in the diabetic retina offering a unique opportunity to use specific inhibitors to prevent such alterations. In fact *in vivo* PKCζ inhibition was sufficient in our model to restore short-term hyperglycemia induced PKCζ alterations. We assume that outer BRB and photoreceptor degeneration could thereby be prevented in the long-term by such treatment. Obviously, further studies are required to confirm its benefit over the long term.

These results are in agreement with *in vitro* endothelial cell studies previously published demonstrating that TNF**-**α signals, through PKCζ/NF-k B pathway alter tight junction complexes and increase retinal endothelial cell permeability [30]. Titchenell et al. showed that PKCζ inhibitor prevent vascular endothelial growth factor (VEGF) induced blood retinal barrier dysfunction [31]. Similar findings were recently reported in the brain where PKCζ activity mediated hypoxia-induced increase in cortical blood-brain barrier permeability [32].

Several pathways can explain the biphasic activation of PKCζ during diabetes observed in the present study. The ceramide pathway has been shown to be activated in type 2 diabetes patients and their concentration is correlated to the severity of insulin resistance [33]. Moreover it is now well established that while low levels of ceramides activate PKCζ, higher concentrations are on the contrary responsible for its down activation [34], [35]. Furthermore ceramide has been reported to stimulate phospholipase A2 thereby generating arachidonate, one of the first mediators of the inflammatory cascade that can in turn promote or inhibit PKCζ activity depending on its concentration. [36]–[38]. Accumulating evidence stemming from animal and human studies suggest a key role for chronic low-grade inflammation in the development of DR. In this context we have recently shown that PKCζ inhibitor treatment could also prevent the activation of microglial cells within the retina thereby attenuating their proinflamamtory effect supposed to participate substantially in photoreceptor cell death [39].

In conclusion, we have identified PKCζ activity as a potential regulatory target in the early events taking place in the diabetic retina: rupture of the outer limiting barrier and photoreceptor cell death. This window of intervention, before any sign of microangiopathy could prevent irreversible vision loss in the time course of diabetic retinopathy.
